# Rush Medical College

**Published:** 1860-03

**Authors:** 


Rush Medical College.—The exercises of the seventeenth annual commencement of this Institution, were held on the evening of the fifteenth of February. Professor Brainard gave the valedictory address. The number of students in attendance during the course has been something over one hundred; being more than the Institution has averaged in former years; and a more worthy and intelligent class, or one whose members have devoted themselves with greater assiduity to the objects that drew them together, has never before convened within its walls. The course in every respect, has been as useful and quite as satisfactory as that of any previous year.

The whole number of matriculants of the school, up to this time is seventeen hundred, and of graduates, five hundred.—The number of graduates of the class of 1859–60, was thirty-six, whose names and subjects of theses, will appear in the next number of the *Journal*.

